# Clinical, genetic, and histological features of centronuclear myopathy in the Netherlands

**DOI:** 10.1111/cge.14054

**Published:** 2021-09-25

**Authors:** Stacha F. I. Reumers, Corrie E. Erasmus, Karlijn Bouman, Maartje Pennings, Meyke Schouten, Benno Kusters, Floor A. M. Duijkers, Anneke van der Kooi, Bregje Jaeger, Corien C. Verschuuren‐Bemelmans, Catharina G. Faber, Baziel G. van Engelen, Erik‐Jan Kamsteeg, Heinz Jungbluth, Nicol C. Voermans

**Affiliations:** ^1^ Department of Neurology Donders Institute for Brain, Cognition and Behaviour, Radboud University Medical Center Nijmegen The Netherlands; ^2^ Department of Paediatric Neurology Radboud University Medical Center – Amalia Children's Hospital Nijmegen The Netherlands; ^3^ Department of Human Genetics Radboud University Medical Center Nijmegen The Netherlands; ^4^ Department of pathology Radboud University Medical Center Nijmegen The Netherlands; ^5^ Department of Clinical Genetics Amsterdam UMC, University of Amsterdam Amsterdam The Netherlands; ^6^ Department of Neurology Amsterdam University Medical Center Amsterdam The Netherlands; ^7^ Department of Paediatric Neurology Amsterdam University Medical Centers Amsterdam The Netherlands; ^8^ Department of Genetics University Medical Center Groningen, University of Groningen Groningen The Netherlands; ^9^ Department of Neurology, School of Mental Health and Neuroscience Maastricht University Medical Center+ Maastricht The Netherlands; ^10^ Department of Paediatric Neurology, Neuromuscular Service Evelina's Children Hospital, Guy's & St. Thomas' Hospital NHS Foundation Trust London UK; ^11^ Randall Centre for Cell and Molecular Biophysics Muscle Signalling Section, FoLSM, King's College London UK

**Keywords:** *BIN1*, centronuclear myopathy, cohort, *DNM2*, *MTM1*, Netherlands, *RYR1*

## Abstract

Centronuclear myopathy (CNM) is a genetically heterogeneous congenital myopathy characterized by muscle weakness, atrophy, and variable degrees of cardiorespiratory involvement. The clinical severity is largely explained by genotype (*DNM2*, *MTM1*, *RYR1, BIN1, TTN*, and other rarer genetic backgrounds), specific mutation(s), and age of the patient. The histopathological hallmark of CNM is the presence of internal centralized nuclei on muscle biopsy. Information on the phenotypical spectrum, subtype prevalence, and phenotype–genotype correlations is limited. To characterize CNM more comprehensively, we retrospectively assessed a national cohort of 48 CNM patients (mean age = 32 ± 24 years, range 0–80, 54% males) from the Netherlands clinically, histologically, and genetically. All information was extracted from entries in the patient's medical records, between 2000 and 2020. Frequent clinical features in addition to muscle weakness and hypotonia were fatigue and exercise intolerance in more mildly affected cases. Genetic analysis showed variants in four genes (18 *DNM2*, 14 *MTM1*, 9 *RYR1*, and 7 *BIN1*), including 16 novel variants. In addition to central nuclei, histologic examination revealed a large variability of myopathic features in the different genotypes. The identification and characterization of these patients contribute to trial readiness.

## Graphical abstract

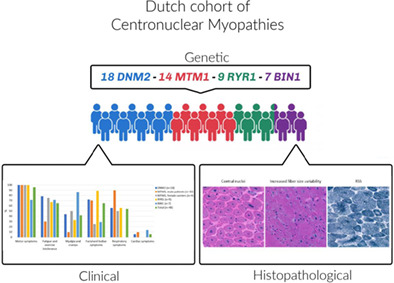

## 1 INTRODUCTION

Centronuclear myopathies (CNM) are a group of congenital myopathies named after the abnormal localization of cell nuclei in the center rather than their normal location at the periphery of skeletal muscle cells.^1^ CNMs are genetically heterogeneous and have been associated with autosomal‐dominant (AD), autosomal‐recessive (AR), and X‐linked inheritance.^2^ Over the past three decades, different genetic causes of CNM have been identified, including variants in *MTM1, DNM2, BIN1*, *RYR1*, and *TTN* and—less frequently—*SPEG1, MYF6, MAP3K20 (ZAK)*.^1^, ^3^, ^4^, ^5^ *MTM1, DNM2*, and *BIN1*, the genes originally implicated in CNM, all encode proteins involved in membrane remodeling and trafficking, while more recently implicated genes such as *RYR1* and *TTN* play important roles in excitation‐contraction coupling and sarcomere assembly, respectively.^5^

As with other congenital myopathies, the most prominent clinical manifestations of CNM are early‐onset muscle weakness, hypotonia, and associated disabilities.^2^ There is substantial variability in the course and degree of functional impairment among the various CNMs.^6^, ^7^, ^8^ Patients may present within the spectrum of the floppy infant syndrome, or with variable degrees of weakness with delayed gross motor milestones, respiratory and/or bulbar involvement.^7^, ^9^, ^10^, ^11^ Presentation is predominantly in infancy and childhood, but some patients do not present until their teens or adolescence with reduced exercise tolerance and mild ptosis, and often remain ambulatory throughout adult life.

The X‐linked form (XL‐MTM) due to *MTM1* gene variants usually gives rise to a severe phenotype in males presenting at birth with marked weakness and hypotonia, external ophthalmoplegia and respiratory failure.^7^, ^12^, ^13^ Patients may never crawl or walk and remain wheelchair‐dependent. XL‐MTM is often lethal in childhood or the teenage years.^14^ Carriers of XL‐MTM are generally considered not to be affected, but several manifesting carriers have been reported in recent years.^15^, ^16^

As a group, patients with *DNM2*‐, *BIN1‐*, and *RYR1‐* variants are generally more mildly affected, but occasionally more severely affected male patients with *RYR1*‐related CNM may mimic XL‐MTM.^2^

Recently, two large studies on *MTM1‐* and *DNM2‐*related CNM have provided essential data concerning their natural history, thus contributing to trial readiness.^13^, ^17^ Also based on these studies, the protocols for current clinical trials in XL‐MTM and *DNM2‐*CNM have included several clinical outcome measures considered to be most discriminative for and/or responsive to change. Another important step towards trial readiness is patient identification and epidemiological data concerning the different genetic subtypes.

To date, CNM epidemiological reports provide limited incidence and prevalence data. A recent integrated model utilizing available literature has been proposed to obtain a better estimate of overall CNM patient numbers by age, causative gene, severity, and geographic region.^18^ This model calculated a CNM incidence higher than the current estimates. Therefore, knowledge on the actual prevalence in a geographically defined region is essential. Our aim therefore was to obtain epidemiological information regarding the Dutch CNM cohort, and to report their clinical, genetic, and histological features. This could also facilitate CNM trial recruitment in the future.

## 2 METHODS

This retrospective, cross‐sectional study was conducted at the Radboudumc Neuromuscular Centre, Nijmegen, in collaboration with the Dutch Neuromuscular Centre. All CNM patients had been referred to our center between 2000 and 2020. The study was approved by the local ethics committee (Protocol 2017‐4022), and all participants or, as appropriate, their parents provided informed consent.

### 2.1 Patients

Inclusion criteria were (1) a (likely) pathogeneous mutation in one of the genes associated with CNM: *MTM1, DNM2, BIN1*, and *RYR1*, and a clinical phenotype of a myopathy; or (2) a clinical or histopathological diagnosis of CNM and genetic confirmation of first degree affected family member. In the first group, histological confirmation was not required since histopathological confirmation is not performed in all cases anymore. We included subjects without genetic confirmation in the second group since some XL‐MTM patients had passed away before the diagnostic availability of genetic testing. Patients were divided into groups per genotype, including a distinction between male patients and female manifesting carriers with an *MTM1* variant.

### 2.2 Data collection

CNM patients of all ages were identified through four routes: (1) the (Paediatric) Neurology Outpatient Clinic at the Radboudumc; (2) the Genetics Department at the Radboudumc; (3) the Dutch Patient Organization Spierziekten Nederland; and (4) (Paediatric) Neurologists of the Dutch Neuromuscular Centre. This is estimated to provide a high coverage (>80%) since the Radboudumc is the national referral center for congenital myopathies and is acknowledged as such by the other Dutch Neuromuscular Centres. Hence, CNM patients are generally referred to the Radboudumc at least once as part of clinical management, for registration and for participation in studies. Clinical data from the patients were stored in our electronic patient file system, and systematically extracted by the researchers (S.R. and D.Z.). Data were pseudonymized and stored in a Castor database.

### 2.3 Data collection, clinical features

We collected information regarding family history, medical history, clinical features, and ancillary investigations. Clinical features were grouped into motor symptoms (signs of delayed gross motor development, muscle weakness, muscle atrophy, and hypotonia), myalgia and cramps (myalgia, muscle cramps, and stiffness), facial and bulbar symptoms (facial weakness, abnormal ocular movements, dysphagia, and dysarthria), respiratory symptoms and cardiac involvement. Age at onset was retrieved from the medical file or estimated based on the history (congenital: 0 years; early childhood: ± 3 years; childhood: ±5 years). Age at diagnosis was determined by the time point where either a histological or suspected genetic diagnosis of CNM was made. Reference values for CK (in IU/L) used in our medical center are ≤710 for neonates, ≤295 for infants, ≤230 for children, ≤270 and ≤ 123 for male and female adolescents, ≤170 and ≤ 145 for men and women.

### 2.4 Data collection, genetic findings

Results of genetic testing previously performed as part of the diagnostic procedure were retrieved from the medical files. In most patients, Sanger sequencing was performed until the introduction of whole‐exome sequencing with muscle panel analysis in 2013.^19^ The variants were classified as pathogenic, likely pathogenic, variant of uncertain significance (VOUS), likely benign or benign, according to the ACMG classification.^20^

### 2.5 Histologic features

Results of muscle biopsy were retrieved from the medical files. Muscle biopsy samples were frozen and stored at −80°C, specimens were processed for routine histological procedures. The majority of samples were processed with several enzyme histochemical staining, including hematoxylin and phloxin (HPhlox), nicotinamide adenosine dinucleotide (NADH), succinate dehydrogenase (SDH), cytochrome C oxidase (COX), Gömöri trichrome, and ATPase 4.2, ATPase 4.6, and ATPase 10.3. All available muscle biopsy slides were reviewed by the pathologist at our center (B.K.) to confirm and/or amend the findings described in the clinical report. We paid particular attention to the following histological features: increased fiber size variability, type I fiber predominance, increased internal and central nuclei (>5%), fatty or connective tissue, nuclear clumps, and radial sarcoplasmic strands (RSS).

### 2.6 Statistical analysis

Data were analyzed using IBM SPSS Statistics software (version 25. Armonk, NY: IBM Corp.). Descriptive statistics used were mean with *SD* (n ± SD) and frequencies with percentages (*n*[%]).

## 3 RESULTS

### 3.1 Patients

We identified 50 patients with a CNM diagnosis in the Netherlands. Two patients with an additional diagnosis of nemaline myopathy were excluded. We retained two patients with a *DNM2* variant and a mixed myopathy—neuropathy phenotype (neurophysiologically and histologically classified).

**FIGURE 1:**
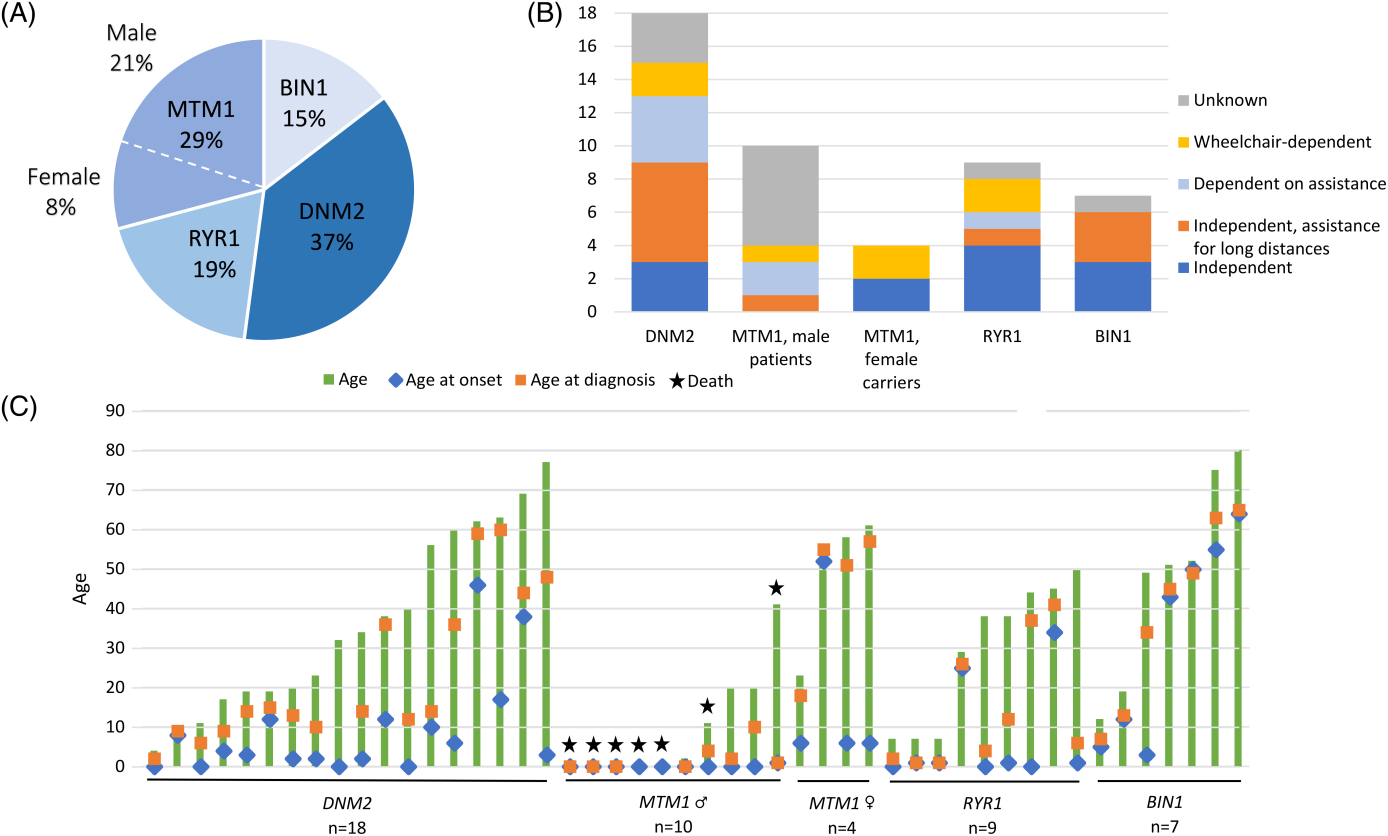
(A) Prevalence of CNM genotypes in the Netherlands. (B) Ambulatory status per genotype. (C) Ages of all patients, including ages at onset and diagnosis [Colour figure can be viewed at wileyonlinelibrary.com]

The most common genotype was *DNM2* (18/48, 37%, 11 families), followed by *MTM1* (14/48, 29%, 9 families) and *RYR1* (9/48, 19%, 8 families). Variants in *BIN1* were least frequent (7/48, 15%, 1 family). There were 10 male *MTM1* patients (10/48, 21%) and four female manifesting carriers (4/48, 8%). Genotype prevalence is shown in Figure 1A.

Seven of the 10 male XL‐MTM patients had passed away (30% survival, mean age at death was 7 ± 15 years). Five out of seven XL‐MTM patients died shortly after birth because of respiratory failure. One patient died at the age of 11 due to respiratory failure after recurrent respiratory tract infections and pneumothorax. The oldest XL‐MTM patient (41 years) passed away because of sudden cardiac failure, this patient was reported previously (III.3).^21^ Survival in the other genotypes was 100%. Of the *DNM2*‐CNM patients, 61% had family members with a diagnosed myopathy; in *MTM1* and *RYR1* patients this was the case in 50% and 44%, respectively. In 13% of the patients stillbirths in the family were reported; these were mainly *MTM1* families. Percentages per genotype are given in Table 1.

**TABLE 1:** Patient characteristics per genotype

	*DNM2* (*n* = 18)	*MTM1,* male patients (*n* = 10)	*MTM1,* female carriers (*n* = 4)	*RYR1* (*n* = 9)	*BIN1* (*n* = 7)	Overall (*n* = 48)
Male sex (%)	8 (44)	10 (100)	0	5 (56)	3 (43)	26 (54)
Age (range), y	36 ± 23 (4–77)	9 ± 14 (0–41)	50 ± 18 (23–61)	29 ± 18(7–50)	48 ± 26 (12–80)	32 ± 24 (0–80)
Age onset, y	9 ± 13	0 ± 0	17 ± 24	7 ± 13	33 ± 26	11 ± 18
Age diagnosis, y	24 ± 19 (*n* = 17)	2 ± 3 (*n* = 8)	45 ± 18	14 ± 16	39 ± 23	22 ± 21 (*n* = 45)
Age at death, y	‐	7 ± 15(*n* = 7)	‐	‐	‐	7 ± 15(*n* = 7)
Delay between onset and diagnosis, y	14 ± 14 (*n* = 17)	2 ± 4 (*n* = 8)	29 ± 24	7 ± 12	6 ± 11	11 ± 14 (*n* = 45)
Family members with neuromuscular disease/symptoms (%)	11 (61)	5 (50)	2 (50)	4 (44)	7 (100)	29 (60)
Stillbirth in family members (%)	1 (6)	2 (20)	3 (75)	0	0	6 (13)
Creatine kinase level, IU/L	199 ± 265 (*n* = 11)	199 ± 144 (*n* = 4)	428 ± 385 (*n* = 4)	117 ± 160 (*n* = 6)	261 ± 218 (*n* = 4)	222 ± 249 (*n* = 29)

*Note*: Values are presented as means with *SD* (*n* ± *SD*) or counts with percentages (*n*[%]).

Abbreviations: IU/L, international units per liter; y, year.

### 3.2 Clinical features

Patient characteristics are summarized in Table 1. Overall mean age was 32 ± 24 years, ranging from 0 to 80 years. All male *MTM1* patients had congenital onset, most *RYR1* and *DNM2* patients had onset in childhood. Age at onset was highly variable for *BIN1* patients. Age at onset in the two *DNM2* patients with a mixed phenotype was 17 and 46 years, respectively. The delay between age at onset and age at diagnosis was only short for male *MTM1* patients (2 ± 4 years) and longest for female *MTM1* carriers (29 ± 24 years). Patient age, including the age at onset and diagnosis, is depicted in Figure 1C.

**FIGURE 2:**
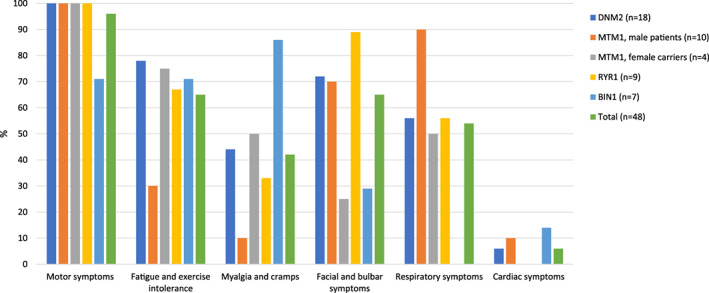
Clinical features per genotype [Colour figure can be viewed at wileyonlinelibrary.com]

Clinical features for each genotype are illustrated in Figure 2, more detailed information is listed in Table 2. Ambulatory status was highly variable (Figure 1B). None of the male *MTM1* patients achieved independent ambulation, and none of the *BIN1* patients were dependent on assistance or a wheelchair. Two patients used disease modifying medicine (nutritional supplements); one *BIN1* patient and one *MTM1* patient used pyridostigmine. In both patients, this had no effect. Two *RYR1* patients used acetylcysteine, but without significant effects. All CNM patients had at least one motor symptom (signs of delayed gross motor development, muscle weakness, muscle atrophy, and hypotonia), except for two *BIN1* patients whose main clinical features were myalgia and muscle cramps. Myalgia and cramps were reported by many *BIN1* patients and female *MTM1* carriers, while fatigue and exercise intolerance were common in all groups of CNM. These symptoms were less prevalent in male *MTM1* and only reported by older XL‐MTM patients. Furthermore, a predominance of facial and bulbar symptoms was reported in *RYR1*, *DNM2*, and male *MTM1* patients, with bulbar symptoms being most prominent in *RYR1* patients. Respiratory insufficiency was most frequently observed in male *MTM1* patients, but occurred also in the other subgroups of CNM except for *BIN1* patients. The prevalence of cardiac involvement was 6 to 14% in *DNM2*, *MTM1*, and *BIN1* patients. Abnormal ocular movement was reported in 12 (mainly *RYR1*) patients. Ptosis was reported in 16 (33%) patients, most frequently in *DNM2* patients (*n* = 9). Disturbed vision due to strabismus or diplopia was reported in 7 patients (15%), mainly in *RYR1* and *MTM1* patients.

**TABLE 2:** Detailed clinical features per genotype

	*DNM2* (*n* = 18)	*MTM1,* male patients (*n* = 10)	*MTM1,* female carriers (*n* = 4)	*RYR1* (*n* = 9)	*BIN1* (*n* = 7)	Overall (*n* = 48)
Any symptoms	18 (100)	10 (100)	4 (100)	9 (100)	7 (100)	48 (100)
Motor symptoms	18 (100)	10 (100)	4 (100)	9 (100)	5 (71)	46 (96)
Delayed gross motor development	12/12	3/3	1/2	6/6	2/3	24/26
Muscle weakness	18/18	7/7	4/4	8/9	5/6	42/44
Muscle atrophy	6/11	4/4	2/3	5/7	3/6	20 /31
Hypotonia	3/4	8/8	0/1	6/6	0/1	17/20
Fatigue and exercise intolerance	14 (78)	3 (30)	3 (75)	6 (67)	5 (71)	31 (65)
Fatigue	14/14	3/4	3/3	4/5	4/4	27/29
Exercise intolerance	10/10	2/2	3/3	3/4	4/4	22/23
Myalgia and cramps	8 (44)	1 (10)	2 (50)	3 (33)	6 (86)	20 (42)
Myalgia	7/9	0/2	1/2	2/5	4/4	14/22
Muscle cramps	3/5	0/1	2/2	0/1	2/2	7/11
Muscle stiffness	1/2	1/1	0/0	1/1	3/3	6/7
Facial and bulbar symptoms	13 (72)	7 (70)	1 (25)	8 (89)	2 (29)	31 (65)
Facial weakness	11/14	7/7	1/2	8/9	1/4	28/36
Abnormal ocular movement	3/11	3/4	1/3	5/7	0/4	12/29
Dysphagia	4/13	3/5	0/1	4/7	0/3	11/29
Dysarthria	2/8	1/2	1/2	3/7	2/3	9/22
Respiratory symptoms	10 (56)	9 (90)	2 (50)	5 (56)	0	26 (54)
Respiratory insufficiency	10/15	9/10	2/3	5/6	0/1	26/35
Cardiac symptoms	1 (6)	1 (10)	0	0	1 (14)	3 (6)
Cardiac involvement	1/12	1/4	0/2	0/4	1/2	3/24
Ambulation						
‐Independent	3/15	0/4	2/4	4/8	3/6	12/37
‐Independent, assistance for long distances	6/15	1/4	0/4	1/8	3/6	11/37
‐Dependent on assistance	4/15	2/4	0/4	1/8	0/6	7/37
Wheelchair‐dependent	2/15	1/4	2/4	2/8	0/6	7/37

*Note*: The blue rows represent a group of clinical features and are a summary of the symptoms listed below. The total counts are shown with percentages (*n*[%]) per genotype. The white rows represent the individual symptoms, the counts are shown as number/total number without missing data.

Other frequently reported symptoms in our cohort of CNM patients were limited range of joint motion in 16 (33%) patients and joint hypermobility in 5 (10%, mainly *RYR1*) patients. Limited range of motion was in 7 (39%) patients due to contractures, in *MTM1, DNM2*, and *RYR1* patients. Shortening of tibialis anterior, Achilles tendons or hamstrings were mainly reported by *DNM2* patients. The remaining patients with a limited range of motion had severe muscle weakness. Four patients (8%) reported paraesthesia. Scoliosis (13/48, 27%) was reported in all genotypes. Reduced reflexes were another frequent feature (27/48, 56%), occurring with all genotypes. In addition to the well‐known clinical myopathy symptoms, bladder complaints including micturition difficulties, recurrent urinary tract infections, and the use of a urine catheter were reported throughout the different subgroups.

### 3.3 Creatine kinase

Creatine kinase (CK) levels were available in 29 CNM patients. Mean CK values are shown in Table 1; 261 ± 218 IU/L in *BIN1*, 117 ± 160 IU/L in *RYR1*, 199 ± 265 IU/L in *DNM2*, 199 ± 144 IU/L in *MTM1* patients, and 428 ± 385 IU/L in female *MTM1* carriers. Of the 29 patients whose CK levels were known, 12 patients had an elevated CK level; 4 *DNM2* patients, 3 *MTM1* carriers, 3 *BIN1* patients, 1 *RYR1* and 1 *MTM1* patient.

### 3.4 Genetic findings

Variants in CNM‐related genes were detected in 45 of 48 participants (94%). Thirteen patients (27%) had a confirmed pathogenic variant, and 16 patients (33%) had a likely pathogenic variant according to the ACMG classification. Three patients (classified as 1 *BIN1* and 2 *MTM1* patients) could not be tested, but were deemed likely to carry a familial pathogenic variant, due to the presence of clinical and histopathological CNM features very similar to those of a genetically confirmed relative. The two *MTM1* patients died postnatally and postmortem genetic testing had not been performed. Only after the death of the third male child in this family, XL‐MTM was diagnosed. The *BIN1* patient was part of the large family, to which all *BIN1* patients in this cohort belong.

Thirty‐six distinct genetic variants were identified in the families (*n* = 29). Twenty of the variants have previously been reported and 16 variants are novel. Genetic variants and their classification identified in the participants are summarized in Table 3, and more detail is listed in Table S1. Ten out of eleven *DNM2* variants were missense variants and one in‐frame deletion was reported. Two *DNM2* variants were identified as *de novo* after segregation analysis (c.596G > A and c.1105C > T) and are associated with a more severe phenotype than the other *DNM2* variants in this study. One *DNM2‐*CNM patient had a somatic mosaicism for the *DNM2* variant (c.1666G > A). Nine variants in *MTM1* were reported, including nonsense, missense, and splice‐site variants, as well as one whole gene deletion. Thirteen different *RYR1* variants were identified, most were occurring in combination with one or two other *RYR1* variants. Three patients only had one *RYR1* variant identified, which we included in this cohort because of evident clinical and/or histological features consistent with CNM. One *BIN1* variant was identified in one family with an autosomal dominant inheritance pattern.

**TABLE 3:** Genetic variants

Patient	Variant DNA	Protein	Variant type	ACMG classification	Reference
*DNM2 (NM_004945.3)*					
1	c.596G > A^a^	p.(Arg199Gln)	Missense	Likely pathogenic	‐
2^b^	c.1058C > G	p.(Thr353Ser)	Missense	Uncertain significance	‐
3	c.1102G > A	p.(Glu368Lys)	Missense	Pathogenic	3
4–6	c.1105C > T^a^	p.(Arg369Trp)	Missense	Pathogenic	3
7–9	c.1393C > T	p.(Arg465Trp)	Missense	Pathogenic	3
10, 11	c.1553G > A	p.(Arg518His)	Missense	Likely pathogenic	18
12	c.1666G > A	p.(Glu556Lys)	Missense	Likely pathogenic	19
13–15	c.1832G > T	p.(Ser611Ile)	Missense	Uncertain significance	‐
16	c.1840G > A	p.(Ala614Thr)	Missense	Uncertain significance	20
17	c.1931_1933del	p.(Gln644del)	In‐frame deletion	Uncertain significance	‐
18^b^	c.2245G > A	p.(Asp749Asn)	Missense	Uncertain significance	‐
*MTM1 (NM_000252.2)*					
19	c.85C > T	p.(Arg29*)	Nonsense	Likely pathogenic	21, 22
20–22	c.686C > A	p.(Ser229*)	Nonsense	Likely pathogenic	‐
23, 24	c.1210G > A	p.(Glu404Lys)	Missense	Uncertain significance	23
25	c.1233G > C	p.(Trp411Cys)	Missense	Uncertain significance	24
26	c.1260 + 2 T > C	r.spl	Splice‐site (in frame)	Uncertain significance	‐
27	c.1261C > T	p.(Arg421*)	Nonsense	Pathogenic	23
28, 29	c.1354‐2A > T	r.spl	Splice‐site (in frame)	Uncertain significance	‐
30	c.1496G > T	p.(Trp499Leu)	Missense	Likely pathogenic	25
31	c‐76‐?_*1548del	p.0	Entire gene deletion	Likely pathogenic	26
*RYR1 (NM_000540.2)*					
32	c.325C > T	p.(Arg109Trp)	Missense	Uncertain significance	27 ‐
	c.5815‐16G > A	r.(spl?)	Splice‐site	Uncertain significance	
33	c.1100G > T	p.(Arg367Leu)	Missense	Uncertain significance	28
34	c.2653C > T	p.(Arg885Cys)	Missense	Uncertain significance	‐
	c.2671_2786 + 34del	p.(Thr891fs)	Frameshift	Likely pathogenic	‐
	c.4405C > T	p.(Arg1469Trp)	Missense	Uncertain significance	5
35	c.10616G > A	p.(Arg3539His)	Missense	Uncertain significance	29 30
	c.2870 + 1G > A	r.spl	Splice‐site (in‐frame)	Uncertain significance	
36	c.10616G > A	p.(Arg3539His)	Missense	Uncertain significance	29 ‐
	c.13033_13067del	p.(Ala4345fs)	Frameshift	Likely pathogenic	
37	c.10616G > A	p.(Arg3539His)	Missense	Uncertain significance	29 31
	c.14804‐1G > A	r.spl	Splice‐site	Likely pathogenic	
38	c.4454G > A	r.(spl?)/p.(Ser1485Asn)	Splice‐site	Uncertain significance	‐
	c.9103G > C	p.(Glu3035Gln)	Missense	Likely pathogenic	‐
39, 40	c.12083C > T	p.(Ser4028Leu)	Missense	Likely pathogenic	32
*BIN1 (NM_139343.2)*					
41–45	c.53 T > A	p.(Val18Glu)	Missense	Pathogenic	33

Abbreviations: ACMG, American College of Medical Geneticists; VOUS, variants of unknown significance.

^a^ *De novo* variants.

^b^ Patients with a mixed CNM/CMT phenotype.

### 3.5 Muscle histology

**FIGURE 3:**
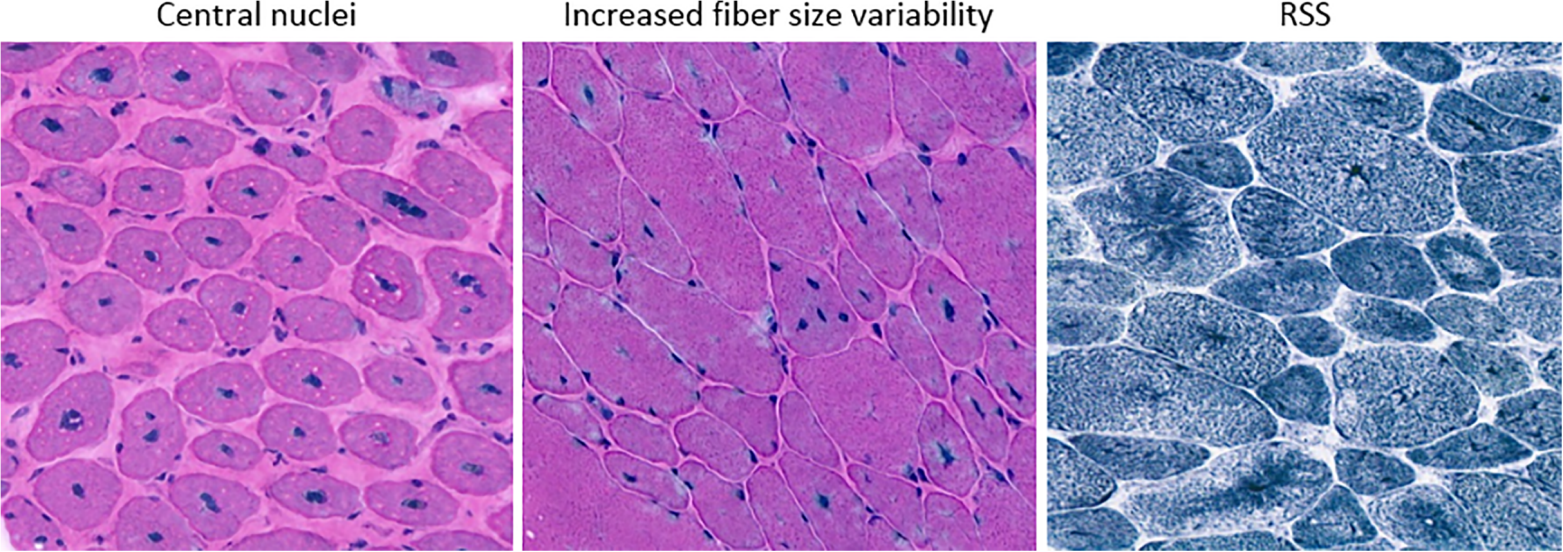
Histopathological features observed in our cohort; central nuclei, increased fiber size variability, and RSS [Colour figure can be viewed at wileyonlinelibrary.com]

Muscle biopsy had been performed in 31 (65%) of 48 patients as part of the routine diagnostic process (M = 16, F = 15). The majority of biopsies were taken from the quadriceps femoris (*n* = 19, 61%), one was taken from the tibialis anterior (2%); the remaining 11 muscle biopsy sites were unknown. Age at biopsy was known for 29 of the patients, with a mean of 29 ± 23 (range 0–66) years. Histological information was extracted from the muscle biopsy reports. Twenty‐three biopsies were reviewed prospectively. Histologic examination revealed frequent internal and central nuclei, in 71% of the muscle biopsies. Increased fiber size variability (17/31, 55%) and type I fiber predominance (13/31, 42%) were also common, although the latter was not observed in female *MTM1* carriers. Fatty or connective tissue was observed in 26% of all muscle biopsies, but not in *BIN1* patients. Nuclear clumps were only reported in *DNM2* and *BIN1* patients and female *MTM1* carriers. Furthermore, radial sarcoplasmic strands (RSS) were frequently present in *DNM2* patients (54%), but only sporadically seen in *RYR1*‐CNM, *BIN1*‐CNM, and female *MTM1* carriers. Core‐like structures were observed in six muscle biopsies, mainly in patients with a *RYR1* variant. In one female *MTM1* carrier, necklace fibers were observed. Typical histopathological features seen in our cohort are displayed in Table 4 and shown in Figure 3.

**TABLE 4:** Histologic findings per genotype

	*DNM2* (*n* = 11)	*MTM1,* male patients (*n* = 5)	*MTM1,* female carriers (*n* = 3)	*RYR1* (*n* = 7)	*BIN1* (*n* = 5)	Overall (*n* = 31)
Increased fiber size variability	6 (55)	1 (20)	3 (100)	5 (71)	2 (40)	17 (55)
Type I fiber predominance	4 (36)	2 (40)	‐	5 (71)	2 (40)	13 (42)
Increased internal nuclei	7 (64)	5(100)	3 (100)	6 (86)	4 (80)	22 (71)
Increased central nuclei	7 (64)	5 (100)	1 (33)	6 (86)	3 (60)	22 (71)
Fatty/connective tissue	4 (36)	1 (20)	2 (67)	1 (14)	‐	8 (26)
Nuclear clumps	2 (18)	‐	3 (100)	‐	2 (40)	7 (23)
RSS						
‐ High degree	3 (27)	‐	‐	‐	‐	3 (10)
‐ Low degree	3 (27)	‐	1 (33)	1 (14)	2 (40)	7 (23)

*Note*: Numbers are presented as counts with percentages (*n*[%]).

Abbreviation: RSS, radial sarcoplasmic strands.

## 4 DISCUSSION

This study describes the clinical, genetic, and histopathological features of a Dutch CNM cohort (*n* = 48). A majority of 37% had a *DNM2* gene variant (*n* = 18, 11 families), 29% had a *MTM1* variant (*n* = 14, 9 families), 19% a *RYR1* variant (*n* = 9, 8 families), and 15% a *BIN1* variant (*n* = 7, 1 family). This nationwide cohort study underlined the wide range of disease severity among the different genotypes and includes data of pediatric and adult patients throughout the life span (0–80 years). Most prominent consistent clinical findings next to muscle weakness were hypotonia, fatigue, and exercise intolerance. Thirty‐six unique variants were identified, including 16 novel variants. Most prominent histological features were by definition frequent internal and central nuclei. Our results detail the clinical, genetic, and histological features of this rare group of congenital diseases in the Netherlands, and give some insights into their prevalence as a basis for future trial readiness.

This is the first study to report the entire cohort of CNM patients in the Netherlands. Similar studies have been previously performed in Denmark and Italy.^22^, ^23^ In these and our current cohort, *DNM2* was the most common genotype. In contrast to the Italian cohort, we did not identify *TTN* mutations. This might be related to the only relatively recent introduction of *TTN* sequencing into clinical diagnostics, and the considerable challenges of variant interpretation in the giant *TTN* gene. Furthermore, we identified a family of seven patients with autosomal dominant *BIN1*‐CNM, which was not observed in the other European cohorts.

The prevalence of CNM in this Dutch cohort differs from the estimated prevalence of CNM in Europe, predicted by the model of Vandersmissen et al.^18^: *MTM1* variants were considered most prevalent (56%), followed by *DNM2* and *RYR1* variants (both 12%), and, least commonly, autosomal dominant *BIN1* variants (4%).^18^ The high prevalence of *DNM2‐*CNM in our cohort might be related to the inclusion of several large *DNM2* families; we included 18 *DNM2*‐CNM patients from 11 families. The wide availability of genetic and prenatal testing in the Netherlands might have contributed to the lower XL‐MTM prevalence,^24^ Dutch policy has a lot of influence on this. Because of the routine ultrasound examination around 20 weeks of pregnancy, termination of pregnancy is more common.^25^ In addition, euthanasia for neonates has been allowed in the Netherlands since 2005.^26^ Moreover, we have performed a retrospective study most likely confounded by selection bias. Therefore, we might not have reported the totality of prevalent patients but rather the majority of them, reaching an estimate of the prevalence in the Netherlands.

We will discuss the most important findings for each genotype in the following section.

### 4.1 *DNM2*‐CNM

Most patients had symptom onset in childhood or adolescence (1–17 years). The three patients with *de novo* variants had an early onset and respiratory insufficiency, as reported in some other patients with *de novo DNM2* variants.^27^ Some *DNM2* variants have been described to be more frequent in late‐onset phenotypes,^23^ however, this was not observed in our cohort. The delay between onset and diagnosis was long (14 ± 14 years), probably related to the late onset, relatively mild symptoms, and slow progression,^9^ and the limited diagnostic options before the era of exome sequencing.

Two patients (patient 2 and 18) were diagnosed with a mixed phenotype of a congenital myopathy and a polyneuropathy, confirmed by electromyography (EMG) and nerve conduction velocity (NCV) studies. In one patient, NCV showed a reduced CMAP amplitude of tibial nerve (0.2 mV). EMG of distal muscles in arms and legs showed signs of denervation, proximal muscles showed signs of both denervation and reinnervation. In the second patient, NCV showed reduced SMAP amplitude of median, ulnar, peroneal, and tibial nerve (2.2; 2.8; 0.8; 0.2 mV). SNAP amplitudes of median and ulnar nerves were also reduced (3.6; 3.8 mV). EMG of distal muscles in arms and legs showed signs of denervation. The genetic variants of these patients are listed in Table 3. *DNM2* plays an important role in mutated cytoskeleton and membrane proteins, both involved in CNM and Charcot–Marie–Tooth disease (CMT). In the CNM phenotype it affects mainly skeletal muscles and in CMT it mainly affects peripheral nerves.^28^ These mixed phenotypes, although not common, have been well‐recognized in *DNM2*‐CNM and might be considered for inclusion in future CNM trials.^29^ Mosaicism detected in one patient has only been reported once,^30^ also with a mild phenotype (no hypotonia, respiratory or feeding difficulties at birth). Two of 11 mutations were *de novo*, which is not uncommon in *DNM2*‐CNM.^27^, ^30^

Connective tissue replacement was the most prevalent muscle biopsy finding in *DNM2* patients. This could be an age effect, since four *DNM2* patients had their muscle biopsy at ages >35 years. Variable degrees of radial sarcoplasmic strands were present in six patients; both features have been described previously.^9^, ^31^

### 4.2 XL‐MTM male patients and female carriers

Male XL‐MTM patients mostly have congenital onset and are severely affected. Cardiac involvement was reported in one male patient in our cohort and not in female carriers, although cardiomyopathies have been previously also described in *MTM1* carriers.^32^ There is also one report of a mildly affected male with XL‐MTM who developed a cardiomyopathy in early adulthood.^33^ Seven patients had passed away during the retrospective study window of 20 years. The severe phenotype in males may be the reason for the only short delay between onset and diagnosis (2 ± 3 years). Fatigue and exercise intolerance were common in all groups of CNM, except for the male *MTM1* patients (30%). This is probably biased by the early death of half of these patients, and also because of their severely reduced mobility. Female manifesting carriers generally have a later onset and a less severe phenotype. This, and the fact that disease manifestation in carriers has been neglected for a long time, has probably contributed to the long diagnostic delay (29 ± 24 years). Our cohort included four affected XL‐MTM carriers, one of them previously published by Biancalana et al.^16^, ^34^ The female phenotype covers almost the full disease spectrum in males, with wheelchair dependency (50%), respiratory insufficiency (50%), and facial and bulbar symptoms (25%, including extraocular muscle involvement).

Nine variants in the *MTM1* gene were identified, including one entire gene deletion which has been reported before.^35^ Type I fiber predominance in muscle biopsies was not reported in female *MTM1* carriers, and nuclear clumps did not occur in XL‐MTM males. In one female *MTM1* carrier, necklace fibers were observed in the biopsy.

### 4.3 *RYR1*‐CNM

Most *RYR1* patients had onset in childhood or adolescence. Findings in the patients with *RYR1*‐CNM were similar to those previously reported in the literature, including joint hypermobility,^36^ facial and bulbar symptoms,^5^, ^10^ absence of cardiac involvement,^5^, ^10^ and normal CK levels.^37^ Thirteen different *RYR1* variants were reported, mainly occurring in combination with one or two other variants. Three patients only had one *RYR1* variant identified. In five patients, additional core‐like structures, which are a common feature in *RYR1‐*related myopathies, were observed in the muscle biopsies.^10^ Nuclear clumps were not observed in any of the *RYR1* patients, RSS only in one patient with a low degree.

### 4.4 *BIN1*‐CNM

All *BIN1* patients in our cohort belonged to one previously reported family with a dominant inheritance pattern.^38^ Family members had a mild phenotype with variable age at onset, with the longest diagnostic delay (31 years) in the index patient. After the first family member was diagnosed, the others soon followed. This might give a biased representation of the diagnostic delay in this patient group. Two genetically confirmed patients in this pedigree did not have any motor symptoms, but had only myalgia and muscle cramps. None of the patients in our cohort was reported to have respiratory insufficiency. The mild phenotype is in line with other dominant cases,^11^, ^39^ whereas recessive *BIN1* cases are generally more severe.^11^

Fatty or connective tissue was not observed in *BIN1* biopsies. In a case report of a severely affected *BIN1* patient, there was an increased amount of connective tissue.^40^ However, this is not known as a typical feature in *BIN1*‐CNM patients.

We included patients with either a histopathological diagnosis of CNM, or suspected genetic diagnosis of mutations in a gene implicated in CNM. Muscle biopsy had been performed in 65% of all patients as part of the routine diagnostic process. The other 35% of patients had a mutation in one of the CNM‐related genes and a clinical phenotype consistent with CNM and/or family members with the same diagnosis and histopathological CNM features. This illustrates how the diagnosis of congenital myopathies is gradually changing with the increasing availability of genetic testing. We included patients with variants of uncertain significance and patients with only one *RYR1* variant identified in our cohort, because we assume all‐encompassing (clinical diagnosis, family history/diagnosis, and possibly biopsy) that this is the cause of the clinical manifestation.

Central nuclei would be expected in all CNM muscle biopsies. However, the muscle biopsy of several patients with a pathogenic variant in one of the CNM genes (*n* = 9) showed no centralized nuclei at all. This is likely to be related to our current sequence of ancillary investigations—first genetic testing and subsequently muscle biopsy^4^, ^41^—in contrast to the historical diagnostic approach where genetic testing was mainly prompted by suggestive features on muscle biopsy. Fatty or connective tissue and nuclear clumps were less common features in our CNM cohort. Nuclear clumps can occur in long‐standing neurogenic or myopathic conditions, but also in other genetic muscle disorders such as myotonic dystrophy type 2 (DM2).^42^, ^43^ Fibrosis and increases in fatty tissue have previously been reported in *DNM2*‐ and *RYR1*‐CNM with varying frequency and severity. In our cohort, fibrosis was observed in 36% of our *DNM2* patients.^30^, ^41^ Although RSS were described in previous studies focusing on CNM patients with mainly autosomal‐dominant inheritance, we found this feature in only 10 of our biopsies, including six biopsies from patients with a *DNM2* mutation.^41^ The range of patient age when the muscle biopsy was performed was wide (0–66 years), with a likely effect on the observed variability of features, as some abnormalities in the muscle are not always visible at a younger age and may only develop over time. Other features, for example type I fiber predominance and the presence of connective tissue that are known to increase as part of the aging process,^44^ may be more prominent in biopsies taken at an older age.

A limitation of this study is the retrospective study design. Data were collected by medical chart review, preventing a more detailed description of the phenotype of this cohort. Another constraint is the small size of the different genetic subgroups, resulting in difficulties in making comparisons between the different genotypes and with regards to the wider applicability of our findings. In addition, previously reported genotype–phenotype variability and intrafamilial variability have to be taken into account.^38^, ^45^, ^46^ The next step will be to assess these patients prospectively to collect natural history data. A recent natural history study in Belgium and France focusing on CNM patients with a *DNM2* mutation has provided reliable natural history data and sensitive outcome measures.^17^ Results of this have contributed to the design of the ongoing phase I/II trial (ClinicalTrials.gov Identifier: NCT04033159), investigating a new medicine named DYN101 in patients with *DNM2* and *MTM1* mutations.^47^

In conclusion, to our knowledge, this is the first detailed study in the Netherlands to report the complete identified Dutch CNM cohort. The identification and characterization of these patients contributes to trial readiness.

## CONFLICT OF INTEREST

The authors declare to have no conflicts of interest.

### PEER REVIEW

The peer review history for this article is available at https://publons.com/publon/10.1111/cge.14054.

## Supporting information


**Appendix** **S1:** Supplementary Information

## Data Availability

The data that support the findings of this study are available from the corresponding author upon reasonable request.
