# Supplementary material for: Clinical, genetic, and histological features of centronuclear myopathy in the Netherlands
Source: Clin Genet. 2021 Sep 25;100(6):692–702. doi: 10.1111/cge.14054 (PMC9292987; doi:10.1111/cge.14054)
Supplement: Supplementary file 1 — Appendix S1: Supplementary Information [file CGE-100-692-s001.docx]

# Supplement

**Supplementary table 1.** Details of the genetic variants.

| Genotype | Genomic position | Variant DNA | Protein | Variant type | CADD (PHRED) | Freq in gnomAD (%) | ACMG details | ACMG classification | Refer-ence |
| --- | --- | --- | --- | --- | --- | --- | --- | --- | --- |
| *BIN1 (NM_139343.2)* | Chr2(GRCh37):g.127864467A>T | c.53T>A | p.(Val18Glu) | Missense | 25,4 | - | PS3, PM2, PP1 | Pathogenic | [32] |
| *DNM2 (NM_004945.3)* | Chr19(GRCh37):g.10887800G>A | c.596G>A^†^ | p.(Arg199Gln) | Missense | 31 | - | PS2, PM2, PP3 | Likely pathogenic | - |
|  | Chr19(GRCh37):g.10904461C>G | c.1058C>G | p.(Thr353Ser) | Missense | 25,7 |  | PP3 | Uncertain significance | - |
|  | Chr19(GRCh37):g.10904505G>A | c.1102G>A | p.(Glu368Lys) | Missense | 29,3 | - | PS2, PS3, PM2, PP3 | Pathogenic | [3] |
|  | Chr19(GRCh37):g.10904508C>T | c.1105C>T^†^ | p.(Arg369Trp) | Missense | 29,2 | - | PS2, PS3, PM2, PP3 | Pathogenic | [3] |
|  | Chr19(GRCh37):g.10909219C>T | c.1393C>T | p.(Arg465Trp) | Missense | 27,5 | - | PS2, PS3, PM2, PP3 | Pathogenic | [3] |
|  | Chr19(GRCh37):g.10922947G>A | c.1553G>A | p.(Arg518His) | Missense | 26,4 | - | PS4, PM2, PP3 | Likely pathogenic | [18] |
|  | Chr19(GRCh37):g.10930662G>A | c.1666G>A | p.(Glu556Lys) | Missense | 23,7 | - | PS3, PM2, PP3 | Likely pathogenic | [19] |
|  | Chr19(GRCh37):g.10934526G>T | c.1832G>T | p.(Ser611Ile) | Missense | 27,8 |  | PP3 | Uncertain significance | - |
|  | Chr19(GRCh37):g.10934534G>A | c.1840G>A | p.(Ala614Thr) | Missense | 27,5 | 0,000032 | PS3 | Uncertain significance | [20] |
|  | Chr19(GRCh37):g.10935782_10935784del | c.1931_1933del | p.(Gln644del) | In-frame deletion | 24,1 | - | PM2, PP3 | Uncertain significance | - |
|  | Chr19(GRCh37):g.10939910G>A | c.2245G>A | p.(Asp749Asn) | Missense | 18,33 | 0,000400 |  | Uncertain significance | - |
| *RYR1 (NM_000540.2)* | Chr19(GRCh37):g.38934252C>T | c.325C>T | p.(Arg109Trp) | Missense | 28,1 | 0,007800 | PP3 | Uncertain significance | [26] |
|  | Chr19(GRCh37):g.38939431G>T | c.1100G>T | p.(Arg367Leu) | Missense | 25,9 | - | PM2, PP3 | Uncertain significance | [27] |
|  | Chr19(GRCh37):g.38954138C>T | c.2653C>T | p.(Arg885Cys) | Missense | 24,8 | 0,003600 | PM5, PP3 | Uncertain significance | - |
|  | Chr19(GRCh37):g.38954156_38954524del | c.2671_2786+34del | p.(Thr891fs) | Frameshift | 37 | - | PVS1, PM2 | Likely pathogenic | - |
|  | Chr19(GRCh37):g.38955363G>A | c.2870+1G>A | r.spl | Splice-site (in-frame) | 35 | - | PM2, PM4 | Uncertain significance | [29] |
|  | Chr19(GRCh37):g.38968461C>T | c.4405C>T | p.(Arg1469Trp) | Missense | 26,6 | 0,014000 | PP3 | Uncertain significance | [5] |
|  | Chr19(GRCh37):g.38968510G>A | c.4454G>A | r.(spl?) / p.(Ser1485Asn) | Splice-site | 34 | - | PM2, PP3 | Uncertain significance | - |
|  | Chr19(GRCh37):g.38980700G>A | c.5815-16G>A | r.(spl?) | Splice-site | 19,69 | 0,000400 | PP3 | Uncertain significance | - |
|  | Chr19(GRCh37):g.39001402G>C | c.9103G>C | p.(Glu3035Gln) | Missense | 29,8 | - | PS4, PM2, PP3 | Likely pathogenic | - |
|  | Chr19(GRCh37):g.39016132G>A | c.10616G>A | p.(Arg3539His) | Missense | 27,9 | 0,160000 | PP3 | Uncertain significance | [28] |
|  | Chr19(GRCh37):g.39037155C>T | c.12083C>T | p.(Ser4028Leu) | Missense | 24,4 | - | PS2, PM2, PP3 | Likely pathogenic | [31] |
|  | Chr19(GRCh37):g.39056007_39056041del | c.13033_13067del | p.(Ala4345fs) | Frameshift | 22,3 | - | PVS1, PM2 | Likely pathogenic | - |
|  | Chr19(GRCh37):g.39076577G>A | c.14804-1G>A | r.spl | Splice-site | 32 | - | PVS1, PM2 | Likely pathogenic | [30] |
| *MTM1 (NM_000252.2)* | ChrX(GRCh37):g.149764983C>T | c.85C>T | p.(Arg29*) | Nonsense | 32 | - | PVS1, PM2 | Likely pathogenic | [21] |
|  | ChrX(GRCh37):g.149814163C>A | c.686C>A | p.(Ser229*) | Nonsense | 37 | - | PVS1, PM2 | Likely pathogenic | - |
|  | ChrX(GRCh37):g.149826450G>A | c.1210G>A | p.(Glu404Lys) | Missense | 23,5 | - | PM2 | Uncertain significance | [23] |
|  | ChrX(GRCh37):g.149826473G>C | c.1233G>C | p.(Trp411Cys) | Missense | 32 | - | PM2, PP3 | Uncertain significance | [24] |
|  | ChrX(GRCh37):g.149826502T>C | c.1260+2T>C | r.spl | Splice-site (in frame) | 33 | - | PM2, PM4 | Uncertain significance | - |
|  | ChrX(GRCh37):g.149828137C>T | c.1261C>T | p.(Arg421*) | Nonsense | 36 | 0,000550 | PVS1, PS4 | Pathogenic | [23] |
|  | ChrX(GRCh37):g.149828842A>T | c.1354-2A>T | r.spl | Splice-site (in frame) | 34 | - | PM2, PM4 | Uncertain significance | - |
|  | ChrX(GRCh37):g.149831934G>T | c.1496G>T | p.(Trp499Leu) | Missense | 29,4 | - | PS1, PM2, PP3 | Likely pathogenic | [53] |
|  | ChrX(GRCh37)g.149680273_150154664del | c-76-?_*1548del | p.0 | Entire gene deletion | 27 | - | PVS1, PM2 | Likely pathogenic | [25] |

Abbreviations: ACMG = American College of Medical Geneticists; VOUS = variants of unknown significance.

^†^*De novo* variants.
